# Tropomyosin concentration but not formin nucleators mDia1 and mDia3 determines the level of tropomyosin incorporation into actin filaments

**DOI:** 10.1038/s41598-019-42977-2

**Published:** 2019-04-24

**Authors:** Joyce C. M. Meiring, Nicole S. Bryce, Jorge Luis Galeano Niño, Antje Gabriel, Szun S. Tay, Edna C. Hardeman, Maté Biro, Peter W. Gunning

**Affiliations:** 1 Cellular and Genetic Medicine Unit, School of Medical Sciences, University of New South Wales, Sydney, NSW 2052 Australia; 2 Single Molecule Science, School of Medical Sciences, University of New South Wales, Sydney, NSW 2052 Australia; 3 Pharmaceutical Biology, Center for Drug Research, Ludwig-Maximilians-Universität, Munich, Germany

**Keywords:** Actin, Stress fibres

## Abstract

The majority of actin filaments in human cells exist as a co-polymer with tropomyosin, which determines the functionality of actin filaments in an isoform dependent manner. Tropomyosin isoforms are sorted to different actin filament populations and in yeast this process is determined by formins, however it remains unclear what process determines tropomyosin isoform sorting in mammalian cells. We have tested the roles of two major formin nucleators, mDia1 and mDia3, in the recruitment of specific tropomyosin isoforms in mammals. Despite observing poorer cell-cell attachments in mDia1 and mDia3 KD cells and an actin bundle organisation defect with mDia1 knock down; depletion of mDia1 and mDia3 individually and concurrently did not result in any significant impact on tropomyosin recruitment to actin filaments, as observed via immunofluorescence and measured via biochemical assays. Conversely, in the presence of excess Tpm3.1, the absolute amount of Tpm3.1-containing actin filaments is not fixed by actin filament nucleators but rather depends on the cell concentration of Tpm3.1. We conclude that mDia1 and mDia3 are not essential for tropomyosin recruitment and that tropomyosin incorporation into actin filaments is concentration dependent.

## Introduction

The actin cytoskeleton is implicated in practically every type of cellular process. Actin filaments are incredibly versatile, and are used to produce a broad variety of structures together with a diverse assortment of actin binding proteins^1^. Amongst these, tropomyosin helps define the functionality of actin filaments by influencing actin filament stability and engagement with other actin binding proteins and myosin motors, in an isoform dependent manner^2^. Hence specific tropomyosin isoforms are recruited to specific actin structures^3–5^, although specific actin structures contain several different isoforms, such as stress fibres which are known to require Tpm1.6, 1.7, 2.1, 3.1/3.2 and 4.2^6^. However, the mechanism by which specific tropomyosin isoforms are recruited to distinct actin filaments is not known. Given that tropomyosins dictate the properties of an actin filament, elucidating the mechanism of tropomyosin isoform recruitment in mammalian cells is fundamental to understanding the regulation of the actin cytoskeleton.

Previous work with chimeric tropomyosin mutants has shown that tropomyosins don’t have an autonomous targeting signal but rather that the entire tropomyosin protein is responsible for its sorting^7^. Furthermore, tropomyosin localisation may be perturbed via drugs targeting actin assembly^8,9^, suggesting that tropomyosin sorting occurs at the level of actin filament assembly. This hypothesis is consistent with recent findings that tropomyosin and actin are assembled in parallel on mouse salivary granules *in vivo*^10^. There is also currently no evidence for differences in actin isoform preferences for tropomyosin isoforms *in vitro*^11^. However, even if tropomyosin isoforms did have different preferences for actin isoforms, there are not sufficient different actin isoforms to account for the differences observed in tropomyosin sorting^2^.

A mechanism for tropomyosin sorting has been identified in *Schizosaccharomyces pombe*. Switching the localisation of two yeast formins, For3 and Cdc12, resulted in the exchange in localisation of the two tropomyosin variants (amino-terminally acetylated Cdc8 and un-acetylated Cdc8) present in *S.pombe*^12^. We postulated that formin nucleators would also facilitate tropomyosin recruitment to actin filaments in mammals^2^. Stress fibres contain several different tropomyosin isoforms^6,13^, and mDia1 and mDia3 are two formin nucleators that have been implicated in stress fibre formation^14–16^. Therefore, to test the hypothesis of formin-mediated tropomyosin recruitment, we depleted mDia1 and mDia3 in immortalised human fibroblasts (BJeH). This cell line was selected due to its prominent stress fibres and similar levels of tropomyosin isoforms^13^, making them ideal for detecting differences in the recruitment of specific isoforms. However we found that the depletion of neither mDia1 nor mDia3 individually or together disrupted tropomyosin recruitment to actin filaments, suggesting that the system for tropomyosin recruitment in mammalian cells may differ from the system used in yeast cells. In contrast, we demonstrate that in the presence of excess tropomyosin, the amount of tropomyosin-actin co-polymer is not fixed by assembly factors but rather is determined by the cellular concentration of tropomyosin.

## Results

### mDia1 and mDia3 are not essential for tropomyosin recruitment to actin filaments

**Figure 1:**
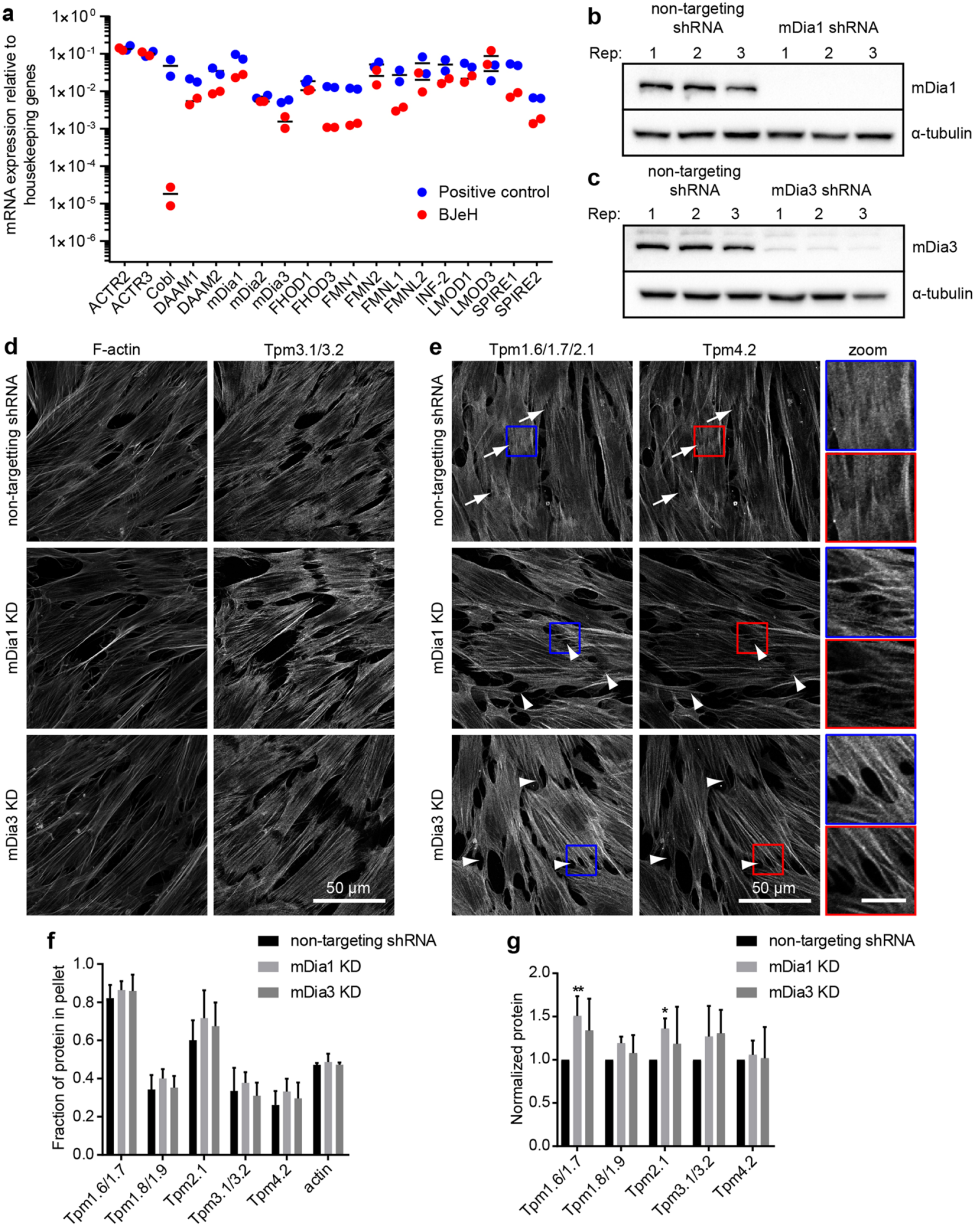
mDia1 and mDia3 are not required for tropomyosin recruitment to actin filaments in cells at steady state. (**a**) Expression of actin nucleators in BJeH cells assessed via qPCR. (**b**,**c**) mDia1 (**b**) and mDia3 (**c**) were depleted separately in BJeH cells via lentiviral-mediated delivery of shRNAmir, knock down was confirmed in 3 different passages via western blotting. (**d**,**e**) non-targeting shRNA, mDia1 KD and mDia3 KD cells were fixed and co-stained for (**d**) F-actin and Tpm3.1/3.2 or (**e**) Tpm1.6/1.7/2.1 and Tpm4.2. mDia1 KD and mDia3 KD cells made poorer cell-cell junctions (stemless arrow heads) than non-targeting shRNA control cells (stemmed arrow heads). Per group a zoomed in image of one junction is displayed on the right. (**f**) Partitioning of tropomyosins and actin with F-actin pellet was measured via a biochemical assay in mDia1 and mDia3 KD cells as well as non-targeting shRNA expressing cells. Graph shows data from 3 independent experiments presented as mean ± SD. (**g**) Protein levels of tropomyosins were measured via western blotting, normalised to α-tubulin and compared between mDia1 KD, mDia3 KD and non-targeting shRNA expressing BJeH cells. Graph shows quantification across 3 independent experiments presented as mean ± SD. **P < 0.01, *P < 0.05. Scale bar shows 10 μm unless stated otherwise.

We established which formins and nucleators are expressed in BJeH cells using qPCR. BJeH cells tested positive for the vast majority of nucleators, including formins mDia1 and mDia3 (Fig. 1a). To deplete mDia1 and mDia3, shRNA sequences against mDia1 and mDia3 as well as a non-targeting control were cloned into miR-E lentiviral expression vectors and transduced into BJeH cells via lentivirus. Transduced cells were selected using puromycin resistance and GFP expression. Knock down of mDia1 and mDia3 was confirmed via Western blotting and measured to be >99% for mDia1 and 91% for mDia3 (Fig. 1b,c). No difference was seen in overall stress fibre morphology (Figs 1d, S1) so to determine if knock down of mDia1 and mDia3 caused the removal of specific tropomyosin isoforms from actin filaments, knockdown cells were stained with antibodies against the major tropomyosin isoforms. No difference was observed in tropomyosin localisation between non-targeting shRNA, mDia1 KD and mDia3 KD cells (Fig. 1d,e). Quantification of fluorescence intensity revealed no difference in the ratio of Tpm3.1 to actin in either control or knockdown cells (Fig. S1). However differences were observed in cell morphology with mDia1 and mDia3 KD cells forming poorer attachments to neighbouring cells (arrows, Fig. 1d). This observation is consistent with recent studies implicating mDia1 in cell-cell junction integrity^17,18^.

Due to the high similarities between tropomyosin isoforms it is impossible to distinguish the individual recruitment of tropomyosins such as Tpm1.6/1.7/2.1 to an actin filament in an immunofluorescence assay. It is possible to measure the population of individual isoforms using a G-actin/F-actin partitioning assay, where the ratio of tropomyosin associated with actin filaments to soluble tropomyosin can be quantified^13^. However, such biochemical assays detected no significant difference in tropomyosin partitioning in mDia1 and mDia3 KD cells (Figs 1e, S1, S4). There was also no significant change in the ratio of G-actin to F-actin in mDia1 or mDia3 KD cells, suggesting that compensation of actin filament nucleation may be occurring in these cells. However, a small but significant increase in high molecular weight tropomyosins Tpm1.6/1.7 and Tpm2.1 was detected in mDia1 KD cells (Figs 1f, S1, S2).

### mDia1 and mDia3 are not essential for recovery of actin-tropomyosin co-polymers after Latrunculin A washout

**Figure 2:**
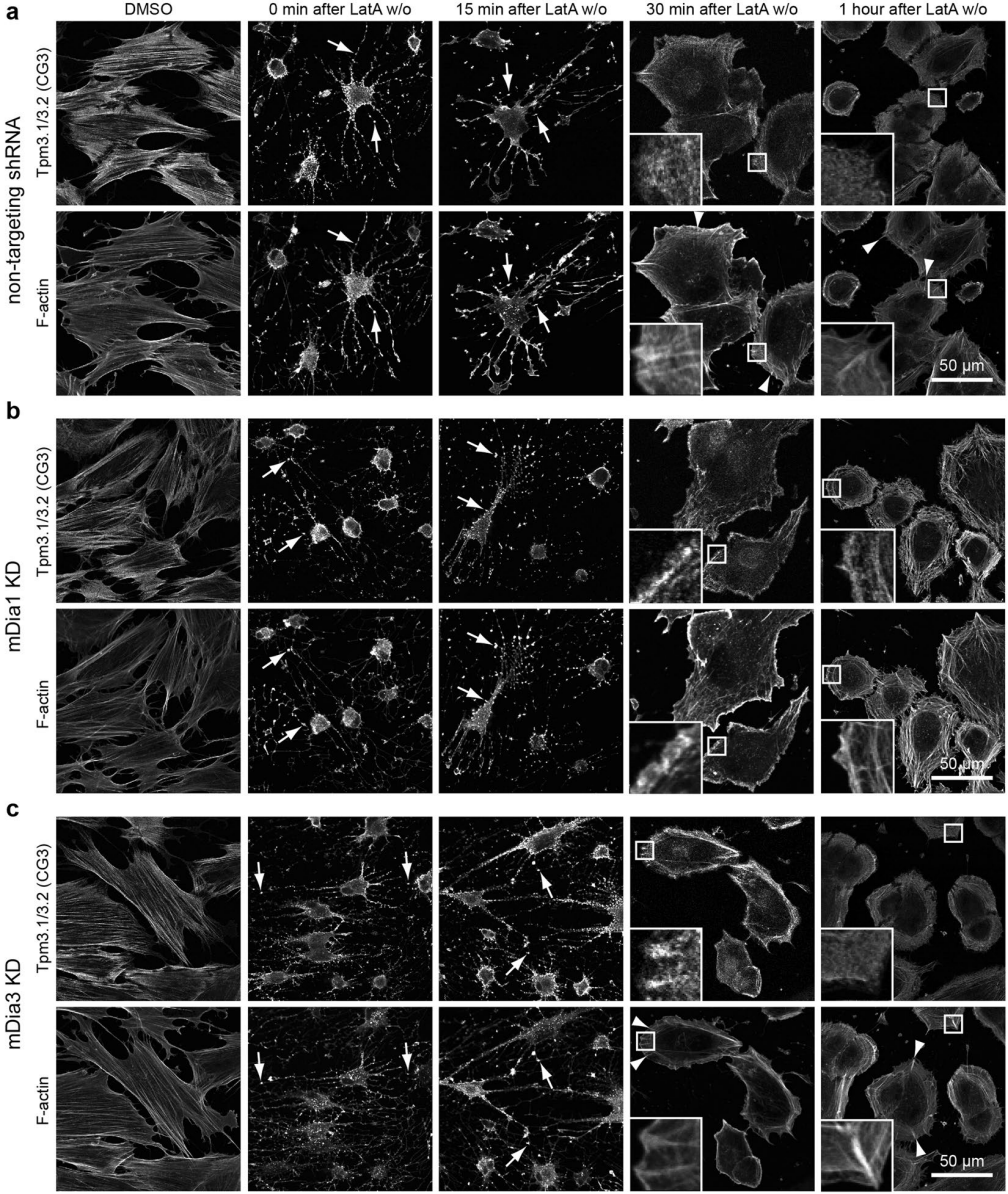
mDia1 and mDia3 are not required to recover Tpm3.1/3.2 containing stress fibre structures after Lat A washout. (**b**) mDia1 KD, (**c**) mDia3 KD and (**a**) non-targeting shRNA transfected BJeH cells were treated with DMSO or LatA and fixed and stained for F-actin and Tpm3.1/3.2 (CG3) at different time points after wash out (w/o). Directly after LatA treatment Tpm3.1/3.2 co-localises with F-actin foci (stemmed arrows). Non-targeting shRNA and mDia3 KD cells show dorsal stress fibres at 30 min and 1 hour after LatA w/o (stemless arrow heads) while mDia1 KD cells do not. Zoomed in images highlights the reduction in dorsal stress fibres in mDia1 KD cells 30 minutes after LatA wash out.

**Figure 3:**
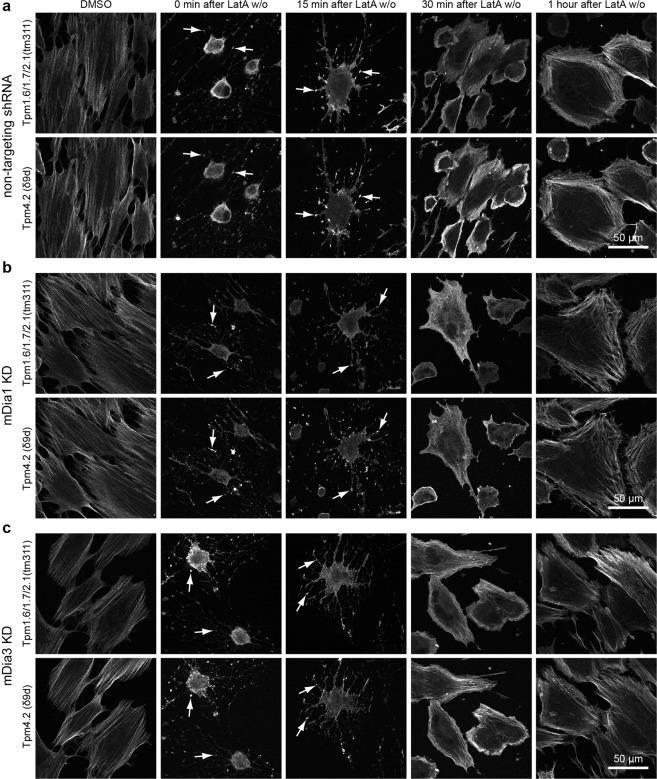
mDia1 and mDia3 are not required to recover Tpm1.6/1.7/2.1 and Tpm4.2 containing stress fibre structures after Lat A washout. (**b**) mDia1 KD, (**c**) mDia3 KD and (**a**) non-targeting shRNA transfected BJeH cells were treated with DMSO or LatA and fixed and stained for Tpm1.6/1.7/2.1 (tm311) and Tpm4.2 (δ9d) at different time points after wash out (w/o). Directly after LatA treatment Tpm1.6/1.7/2.1 and Tpm4.2 co-localises with F-actin foci (arrows).

Initial characterisation of the mDia1 and mDia3 KD cells showed no defects in tropomyosin recruitment, however the actin in these cells was unperturbed and in an established equilibrium. Whilst actin structures are still being turned over in this system, it is difficult to observe the nucleation of new actin filaments in this state. Hence, a Latrunculin A (Lat A) washout assay was used for the purpose of observing *de novo* assembly of the actin cytoskeleton and the roles of mDia1 and mDia3 in this process. Lat A sequesters actin monomers thereby inhibiting assembly and resulting in the disassembly of the actin cytoskeleton^19^. After treatment, Lat A was washed off cells and the actin cytoskeleton allowed to recover^20,21^. Directly after treatment with Lat A cells appear rounded with processes containing foci of Lat A resistant actin filaments that co-localise with Tpm3.1/3.2 (stemmed arrows, Fig. 2). Similarly, Tpm1.6/1.7/2.1 was observed to co-localise in foci with Tpm4.2 (stemmed arrows, Fig. 3). The co-localisation of tropomyosins with these F-actin foci was not surprising given that after 1 μM Lat A treatment, some tropomyosin is still able to associate with actin filaments^13^.

At 15 minutes after washout, cells appear less rounded and have begun to spread out and actin-tropomyosin foci are now situated at the periphery of the cell (Figs 2, 3). Linear actin bundles that co-localise with tropomyosin are also visible in some cells at this time point (15 min after LatA w/o, Figs 2, 3). 30 minutes after washout, cells have spread out and show a concentration of F-actin all along the periphery of the cell (30 min after LatA w/o, Fig. 2). Non-targeting shRNA control cells and mDia3 KD cells have begun to form transverse arcs (30 min after LatA w/o, Figs 2a,c, 3a,c). Meanwhile at the same time point, actin in mDia1 KD cells appears highly disordered with actin filaments or bundles radiating inward from the cortex in different directions (30 min after LatA w/o, Figs 2b, 3b). Antibody staining demonstrates that the disorganised actin structures in the mDia1 KD cells 30 minutes after Lat A washout are positive for the same tropomyosins present in transverse arcs (30 min after LatA w/o, Figs 2b, 3b). Furthermore, mDia1 KD cells showed an absence of dorsal stress fibres at 30 minutes and 1 hour after Lat A washout, unlike non-targeting shRNA control cells and mDia3 KD cells (zoomed in insets and arrowheads, Figs 2, S2). This was consistent with the finding that mDia1 drives dorsal stress fibre assembly^14^.

1 hour after LatA washout, F-actin is no longer enriched at the cell periphery but localises primarily in the form of actin bundles in transverse arcs (1 hour after LatA w/o, Fig. 2). Transverse arcs in non-targeting shRNA and mDia3 KD cells are parallel, homogeneous and situated close to one another while transverse arcs formed by mDia1 KD cells are thicker, less homogeneous, not as parallel to one another and with larger gaps in between arcs (1 hour after LatA w/o, Fig. 2). Despite the stress fibre organisation defects observed in mDia1 KD cells, mDia1 and mDia3 knock down cells both showed no impairment in tropomyosin recruitment during recovery of actin structures after Lat A treatment, suggesting that neither formin is required for tropomyosin isoform recruitment.

### Simultaneous depletion of mDia1 and mDia3 does not perturb tropomyosin recruitment

**Figure 4:**
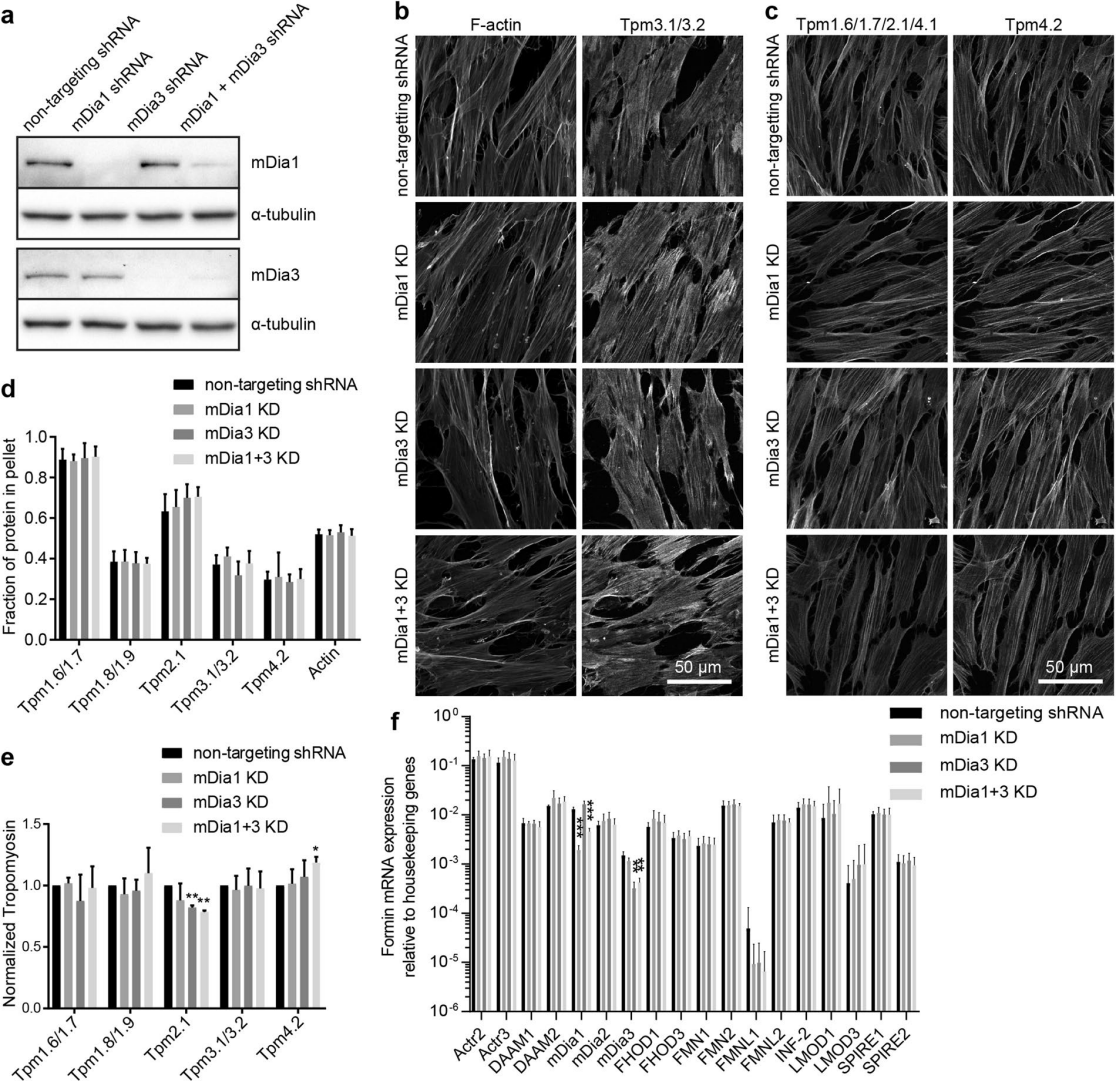
Dual knock down of mDia1 and mDia3 has no significant impact on tropomyosin recruitment or elicit significant compensation from other nucleators. (**a**) mDia1 and mDia3 were depleted separately or simultaneously in BJeH cells via shRNA, knock down was confirmed in via western blotting, using α-tubulin as a loading control. (**b**,**c**) non-targeting shRNA, mDia1 KD, mDia3 KD and mDia1 + mDia3 KD cells were fixed and co-stained for (**b**) F-actin and Tpm3.1/3.2 or (**c**) Tpm1.6/1.7/2.1 and Tpm4.2. (**d**) Partitioning of tropomyosins and actin with F-actin pellet was measured via a biochemical assay in mDia1 KD, mDia3 KD and mDia1 + mDia3 KD cells as well as non-targeting shRNA expressing cells. Graph shows data from 3 independent experiments presented as mean ± SD. (**e**) Protein levels of tropomyosins were measured via western blotting, normalised to α-tubulin and compared between mDia1 KD, mDia3 KD and non-targeting shRNA expressing BJeH cells. Graph shows quantification across 3 independent experiments presented as mean ± SD. (**f**) A qPCR screen was performed to test for compensation from other nucleators in mDia1 KD, mDia3 KD and mDia1 + mDia3 KD cells, with non-targeting shRNA expressing cells as a control. Graph shows results from 3 independent experiments expressed as mean ± SD. ***P < 0.001, **P < 0.01, *P < 0.05.

It is possible that knock down of individual formins may elicit a compensating response in these cells. A double knock down cell line was therefore produced in parallel with individual mDia1 and mDia3 knock down controls as well as a non-targeting shRNA control. Knock downs were confirmed via Western blot (Figs 4a, S5). Knock down cell lines were once again characterised via immunofluorescence and mDia1 + mDia3 double knock downs showed no obvious defects in tropomyosin recruitment (Fig. 4b,c, S5). Double knock down cells also showed no significant difference in tropomyosin partitioning (Fig. 4d). Unlike experiments with the previous mDia1 KD cell line (Fig. 1g), in this second round of experiments there was no significant increase in Tpm1.6/1.7 and Tpm2.1/4.1 with mDia1 KD (Fig. 4e). Since the result could not be replicated in the second generated mDia1 KD cell line, it is not possible to declare with confidence that tropomyosin levels are altered with mDia1 KD. Meanwhile, unlike the first round of experiments (Fig. 1g), in this second round a small statistically significant decrease in Tpm2.1 was detected in the mDia3 KD cells as well as the mDia1 + mDia3 double knock down cells (Fig. 4e). However, this result was not observed in the previous round of experiments. Similarly, the small statistically significant increase in Tpm4.2 observed in mDia1 + mDia3 double knock down cells was not observed in the first experiment (Figs 1g, 4e). It is possible that this difference may be partially attributed to slight differences in knock down levels since mDia1 KD was >99% in the first round and 98% in the second round, meanwhile mDia3 KD was 91% in the first round and 95% in the second.

The data suggest that neither mDia1 nor mDia3 are required for tropomyosin recruitment and that mDia1 and mDia3 also do not engage in compensation, since the phenotype of mDia1 + mDia3 KD cells was no more dramatic than individual mDia1 or mDia3 KD. Since no substantial effect was observed on F-actin levels or tropomyosin recruitment, it is possible that this could be due to compensation from other formin nucleators. Compensation was tested by performing a qPCR nucleator screen as before, this time comparing non-targeting shRNA control cells with mDia1, mDia3 and mDia1 + mDia3 knock down cell lines. While mDia1 mRNA was reduced in mDia1 KD and mDia1 + mDia3 KD cells, and mDia3 mRNA was reduced in mDia3 and mDia1 + mDia3 KD cells respectively, mRNA for the other nucleators tested was not significantly different between cell lines (Fig. 4f).

### Tropomyosin concentration drives incorporation into filaments

We have previously shown that the majority of Tpm3.1/3.2 in human fibroblasts is present in the cytosol and that it is present at a concentration well above that required to drive all the Tpm3.1/3.2 into co-polymers with actin^13^. If a nucleator is determining the amount of Tpm3.1/3.2 co-polymerised with actin it would be expected that reduction of the Tpm3.1/3.2 level by half would not change the amount of Tpm3.1/3.2-actin co-polymer. In other words, the concentration of cytosolic Tpm3.1/3.2 would have no impact on polymeric Tpm3.1/3.2 if an actin nucleator is responsible for assembly of Tpm3.1/3.2-actin co-polymers. We have used Tpm3.1/3.2 hemizygous mouse embryo fibroblast (γ9d +/−) to address this question.

**Figure 5:**
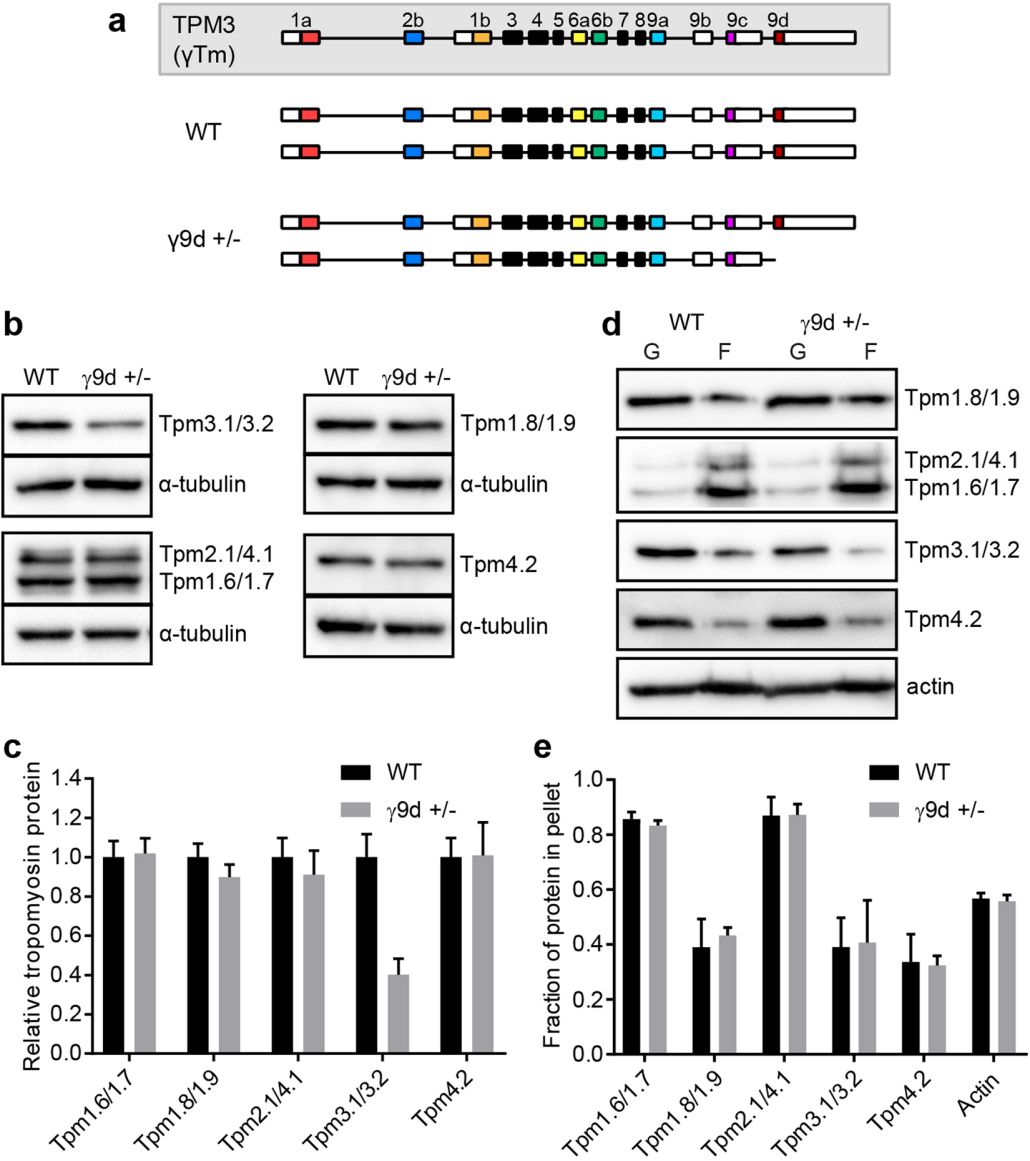
Tpm3.1 recruitment to actin filaments is concentration dependent. (**a**) Genotypes of WT and γ9d +/− MEFs. (**b**,**c**) Tropomyosin protein levels were compared between WT and γ9d +/− MEFs via western blotting. (**b**) Representative tropomyosin blots with α-tubulin as a loading control and (**c**) quantification across 4 independent experiments presented as mean ± SD. (**d** and **e**) Partitioning of tropomyosins and actin with F-actin pellet was measured via a biochemical assay in WT and γ9d +/− MEFs. G indicates globular actin (soluble cytosol fraction), F indicates filamentous actin fraction (pellet). (**d**) Representative blots and (**e**) Quantification across 3 independent experiments presented as mean ± SD. ***P < 0.001.

Hemizygous γ9d +/− Mouse Embryonic Fibroblasts (MEFs) were compared with WT MEFs (Fig. 5a) and confirmed to produce half the amount of Tpm3.1/3.2 whereas the levels of the other tropomyosins were unchanged (Figs 5b,c, S4). However, the partitioning between the cytosol and actin filaments was unchanged for Tpm3.1/3.2 and all other isoforms in the γ9d +/− MEFs (Figs 5d,e, S4). Thus, when the level of Tpm3.1/3.2 is reduced by half, the level of Tpm3.1/3.2 in both the cytosol and in the actin pellet is reduced by half. Depolymerisation of actin filaments with Latrunculin A results in an increase of actin and all tropomyosin isoforms in the cytosol fraction of both WT and γ9d +/− MEFs in an actin partitioning assay confirming that Tpm3.1 levels in the pellet are actin dependent as previously observed in human fibroblasts (Figs S3, S7)^13^. We conclude that cytosolic Tpm3.1/3.2 reflects an equilibrium reaction that allows these tropomyosins to associate with actin filaments and is consistent with both *in vitro* and *in vivo* studies demonstrating that Tpm3.1/3.2 cycles on and off actin filaments^22,23^.

## Discussion

Tropomyosin isoforms are sorted to different actin structures^3–5^, however the mechanism of their recruitment in mammalian cells remains unknown. Recent *in vivo* work in mice shows that unlike Myosin II, tropomyosin closely follows actin enrichment on salivary granules, suggesting that tropomyosin associates with actin filaments during polymerisation or quickly after their formation^10^. This finding is consistent with reports that tropomyosin recruitment can be perturbed by actin-targeting drugs suggesting that tropomyosin recruitment occurs during actin filament assembly. Tropomyosin recruitment is mediated by formin nucleators in yeast^12^. Formin nucleators hence make good candidates for tropomyosin recruitment in mammalian cells.

Despite mDia1 KD showing an effect on stress fibre recovery after Lat A washout (Figs 2b, 3b), mDia1 KD cells did not display any marked differences in tropomyosin recruitment (Fig. 1d–g). This result suggests that mDia1 is not required for tropomyosin recruitment but rather plays a role in actin bundle organisation. Interestingly, unlike mDia2, mDia1 does not have the capacity to act as a cross-linker directly^24^, suggesting that the effect of mDia1 on actin bundle organisation may be indirect. Meanwhile mDia3 KD cells displayed no substantial difference in tropomyosin levels or recruitment, nor in recovery of actin structures after Lat A washout compared to non-targeting shRNA transfected cells. This suggests that mDia3 is also not critical for tropomyosin recruitment. This indicates that mDia1 and mDia3 are not essential for tropomyosin recruitment to actin filaments and that in mammalian cells the mechanism for tropomyosin recruitment may not be as simple as one formin nucleator for each tropomyosin isoform.

While the results of this study do not support a simple relationship between formin and tropomyosin isoform, it is not yet possible to reject the hypothesis. It is possible that tropomyosins can be recruited to actin filaments nucleated by more than one formin nucleator, in which case knocking down an individual formin will not prevent tropomyosin from being recruited. This study tested 2 formins and mammals have another 13 more. Future studies should hence deplete entire formin families to rule out redundancy between formins in their capacity to recruit tropomyosins.

It is also possible that other nucleation factors and co-factors may impact tropomyosin recruitment. Indeed, the Leiomodin (Lmod) family of tropomyosin-binding actin nucleators has been suggested to nucleate tropomyosin-associated actin filaments^25^. However, whether Lmods are capable of recruiting specific tropomyosin isoforms in cells remains to be determined. Furthermore, *in vitro* work suggests that tropomodulins, tropomyosin-binding actin-capping proteins, show some specificity for tropomyosin isoforms^26^, however the contribution of tropomodulins in tropomyosin recruitment has yet to be tested in cells.

In contrast, the absolute role of actin nucleators in determining tropomyosin recruitment is not consistent with the observation that cellular concentrations of Tpm3.1/3.2 are a key determinant in tropomyosin recruitment (Fig. 5). This is consistent with previous studies in which overexpression of tropomyosin isoforms resulted in an increase in stable actin filaments^5,27–30^; indicative of tropomyosin concentration determining tropomyosin incorporation into filaments. Interestingly, concentration has also been identified as a key determinant of tropomyosin incorporation into actin filaments in yeast^31^. Since formin nucleators regulate tropomyosin recruitment in yeast^12^, this suggests that the concentration dependency of tropomyosin incorporation in actin filaments is not incompatible with a formin mediated recruitment mechanism and indicates formins may not determine absolute filament levels. Previous work has also indicated that Tpm3.1/3.2 cycles on and off actin filaments^22,23^. It is therefore possible that soluble Tpm3.1/3.2 is required to generate equilibrium conditions conducive to maintaining Tpm3.1/3.2 associated actin-tropomyosin co-polymers.

In summary, our work shows that mDia1 and mDia3 are not required for tropomyosin recruitment and that mDia1 and mDia3 also do not engage in any compensatory behaviour related to tropomyosin recruitment. However, we find evidence for Tpm3.1/3.2 association with actin filaments being driven by tropomyosin concentration rather than there being a mechanism determining the quantity of Tpm3.1/3.2 associated actin filaments produced in the presence of excess Tpm3.1/3.2. Future studies may need to deplete entire formin families to rule out redundancy between formin nucleators in their capacity to recruit tropomyosin.

## Methods

### Cell culture

BJeH, MEF and HEK293T cells were cultivated in DMEM (Sigma) with 10% FBS (Gibco) at 37 °C in a humidified chamber with 5% CO_2_. For latrunculin washout assays, cells were incubated with 1 µM Lat A for 2 hours, washed once with cell growth media, then allowed to recover for specified times in cell growth media.

### Antibodies

The primary antibodies used in this study are listed as follows: Mouse anti-Tpm3.1,3.2 (CG3, 1:250 for IF, 1:1000 for WB)^32^; Mouse anti-Tpm1.6,1.7,2.1 (Tm311, 1:200 for IF, 1:500 for WB, Sigma Aldrich Cat#T2780); Mouse anti-α-actinin (BM-75.2, 1:500 for IF, 1:2000 for WB, Sigma Cat#A5044); Rabbit anti-Tpm4.2 (δ/9d (2009), 1:50 for IF, 1:500 for WB)^33^; Sheep anti-Tpm1.8,1.9 (α/1b, 1:500 for WB)^33^. Secondary antibodies used for immunofluorescence were: Alexafluor-488 conjugated Goat anti-mouse (1:500, Invitrogen Cat#A-11001), Alexafluor-568 conjugated Goat anti-mouse (1:500, Invitrogen Cat#A-11004); Alexafluor-647 conjugated Donkey anti-rabbit (1:500, Invitrogen Cat#A-31573) and Atto 647N conjugated phalloidin (1:1000, Atto Tec Cat#AD 647N-82). HRP conjugated secondary antibodies used for Western blots were: Donkey anti-rabbit IgG (1:5000, GE Healthcare Cat#NA934), Donkey anti-sheep IgG (1:5000, Santa Cruz Cat#SC-2473), Rabbit anti-mouse IgG (1:10,000, Abcam Cat#ab97046).

### Producing the formin shRNA lentiviral plasmids

miR-E, an optimised version of the miR-30 backbone was used to express the anti-formin RNA interference sequences^34^. Both sequences were designed and verified by Sigma and had a mean knock down level greater than 0.9. The desired target sequences were then put together as follows: 5′mi-RE (TGCTGTTGACAGTGAGCG), sense target sequence converted from RNA to DNA, loop sequence (TAGTGAAGCCACAGATGTA), anti-sense target sequence converted from RNA to DNA, 3′miR-E (TGCCTACTGCCTCGGA). The resulting shRNA sequences were (5′-3′): TGC TGT TGA CAG TGA GCG CGC CCA GAA TCT CTC AAT CTT TTA GTG AAG CCA CAG ATG TAA AAG ATT GAG AGA TTC TGG GCT TGC CTA CTG CCT CGG A for mDia1; TGC TGT TGA CAG TGA GCG ACC TAC AAA GAA GAA AGT GAA ATA GTG AAG CCA CAG ATG TAT TTC ACT TTC TTC TTT GTA GGC TGC CTA CTG CCT CGG A for mDia3. These sequences were subsequently cloned into the SGEP miRE flanked lentiviral expression vector using the *Xho*I and *Eco*RI restriction sites.

### Lentivirus Production

Lentivirus was produced in HEK293T cells by transfecting 3 plasmids: The miRE SGEP lentiviral expression plasmid (containing shRNA against protein of interest) and 2 viral packaging protein vectors, psPAX2 and pMD2.G. This was done using Polyethylenimine (PEI), as follows. Molecular grade PEI (Max molecular weight 4000, Polysciences) was diluted in dH_2_O to 10 mg/mL to make 50 × stocks (stored at −80 °C). 200 μL 50 × PEI stock was mixed with 70 μL 1 M NaOH to neutralise pH, topped up to 10 mL with dH_2_O, and filter sterilised using a 0.22 μm filter. The following quantities of DNA were mixed in the bottom of a 15 mL tube together with 500 μL sterile 0.9% NaCl: 4 μg lenti expression plasmid, 4 μg psPAX2, 2 μg pMD2.G. 60 μL of the pH neutralised, filtered PEI was added to each DNA mix, the mixture was then vortexed at low speed for 10 seconds and incubated at RT for 30 minutes. Meanwhile 293T cells were trypsinised and normalised to 7 × 10^6^/mL. After DNA/PEI incubation 1 mL of cells was added, mixed very gently and incubated at RT for another 5 minutes. 8 mL of standard cell culture media (DMEM + 10% FBS) was then added to the DNA/cell mix and cells were plated on petri dishes. Petri dishes were kept at 37 °C, 5% CO_2_ for 72 hours, without any change of media after 24 hours. After 72 hours cell media was harvested and virus was purified using a Lenti-X concentrator virus purification kit (Clontech). The virus pellet was diluted in cell culture media and stored at −80 °C until use.

### Generating Formin Knock Down Cell Lines

BJeH cells were plated on petridishes and transduced with lentivirus when cells reached 20% confluency. 48 hours after lentiviral transduction cell media was replaced with media containing 4 μg/mL puromycin to select for transduced cells. Media was changed back to standard media after 72 hours of exposure to puromycin. A plate with untransduced cells were used as a control to indicate at what point the vast majority of untransduced cells were dead. 2 weeks post transduction cells were sorted via flow cytometry for GFP and the only the top 5% most fluorescent cells were retained. These cells were then expanded and used for experiments. In order to produce the mDia1/3 double knock down cell line, cells were transduced with both the mDia1 and mDia3 lentivirus and selected with puromycin as above. Cells were sorted for the top 2% most green fluorescent cells rather than the top 5%.

### qPCR

Primers (Table 1) were diluted to 10 μM (1 in 10) and cDNA was diluted to 10 ng/μL (1 in 100), both in DNAse free water. Reactions were performed in 96 well PCR plates with the following mix per well: 7.5 μL SYBR Green (iQ SYBR Green Supermix, Bio Rad), 0.75 μL each of forward and reverse primers, 1 μL DNAse free water and 5 μL diluted cDNA. Plates were centrifuged prior to running inside a Stratagene Mx3000p qPCR machine (Agilent Technologies) with the following cycle conditions: 10 min at 95 °C followed by 40 cycles of: 15 seconds at 95 °C, 30 seconds at 55 °C and 30 seconds at 72 °C.

**Table 1:** Primers used for qPCR.

Primer name	Sequence (5′-3′)
ACTR2 Forward	TACTTCTGTTGCGAGGATAC
ACTR2 Reverse	TCCATCTGGGAGTGTATAAG
ACTR3 Forward	CAGAGTTTGCTAATCCAGAC
ACTR3 Reverse	GGACAATATTCTTGTAGAGAGG
COBL Forward	AAAGGTATCTCTTGGGTCAC
COBL Reverse	CTTCCTGTTCTCCTCCTTATC
DAAM1 Forward	AACGACTTGGATAAAAGCAC
DAAM1 Reverse	GAAGTCTAAAGTCCAAACTCTC
DAAM2 Forward	AGGCAACTTCATGAACAAAG
DAAM2 Reverse	GAGAGATGTTTCTGTCGATG
mDia1 Forward	ACAAGTTTGGAATCAAGACC
mDia1 Reverse	ATACAAAGAGCAGAAAGCAG
mDia2 Forward	AGGAAGTCTTAAGAGAAGCC
mDia2 Reverse	TGTGACAAGTCTCTCATCTG
mDia3 Forward	CTGGTGTGATGGATAATCTTC
mDia3 Reverse	GGATGATTTACCACTGGATTC
FHOD1 Forward	CCAGAGCTACATCCTTAGAG
FHOD1 Reverse	ATTCTACAAACACCAACAGC
FHOD3 Forward	AAGACGTTATCAGGACTACC
FHOD3 Reverse	CTTTCTTGTTCAAGTGCCTC
FMN1 Forward	AAGATTGAAAATGGCTCAGG
FMN1 Reverse	CTAGCAGTGGGATTGATTTTC
FMN2 Forward	AAAGAGACTCCAGTACTTCAC
FMN2 Reverse	AGAGATAGGTTTCTTTCTCTCC
FMNL1 Forward	TCCAGACTAAGTTCCGAATG
FMNL1 Reverse	CAAAATCACTCATGTCTAGCTC
FMNL2 Forward	AGTATGGTTTCAACATGGTC
FMNL2 Reverse	TTCTGGGATTCTTGTTGTTC
INF-2 Forward	AATTTAAGTGCTCCAACGAG
INF-2 Reverse	TTTTCAATCTCGTGCTTCTC
LMOD1 Forward	GAGATGTCCATGGATGAAAG
LMOD1 Reverse	TTTTCTTTAAACCACCCCTC
LMOD3 Forward	GGAAAAGACTCAAGAAGAGC
LMOD3 Reverse	TCACCATCATCTTCTCCTTC
SPIRE1 Forward	AGAAAACTGAAACCAACTCC
SPIRE1 Reverse	GCTAATCTGCTACGTCTAATC
SPIRE2 Forward	AAGAAATTTGGACACATCCC
SPIRE2 Reverse	CTTGCAGAGACTGAAAGATG

### Measuring tropomyosin and actin levels

For each cell line, lysates were collected from 3 different passages. Samples were run on a 10% SDS PAGE gel and transferred to a PVDF membrane using the Trans-Blot Turbo (Biorad) transfer system, then probed with specific anti-tropomyosin antibodies and developed as described above, and normalised to α-tubulin. Densitometry was quantified in Fiji (downloaded from https://fiji.sc/).

### Actin and tropomyosin partitioning assay

The partitioning assay was conducted as described previously^13^, using the G-actin/F-actin *In Vivo* Assay Biochem Kit (Cytoskeleton) according to manufacturer’s instructions. Samples were subsequently run on a 10% SDS PAGE gel and probed with specific anti-actin and tropomyosin antibodies as described above. Densitometry and ratios were analysed using Fiji and Excel. For partitioning experiments using cells treated with LatA, 5 µM LatA or DMSO (vehicle control) was added for 1 h prior to harvesting.

### Microscopy and image analysis

Confocal images were obtained on a Leica TCS SP8 DLS, using a 63×/1.4 oil Plan Apo objective. The microscope was equipped with 488 (Argon), 561 (DPSS) and 633 (HeNe) nm excitation lasers and PMT detectors. Images were captured using Leica Application Suite (LAS) acquisition software. All image analysis was performed in Fiji.

### Statistical analysis

All data are presented as mean ± standard deviation and number of experimental replicates is indicated in the figure legends. Data were analysed in Microsoft Excel 2010 and two-sample two-tailed t-tests were used to compare knock down groups with the non-targeting shRNA control. All graphs were produced using Prism (Graphpad Software).

## Supplementary information

Supplementary Data

## Data Availability

The datasets generated and analysed during the current study are available from the corresponding author on reasonable request.
