# Supplementary material for: Tropomyosin concentration but not formin nucleators mDia1 and mDia3 determines the level of tropomyosin incorporation into actin filaments
Source: Sci Rep. 2019 Apr 24;9:6504. doi: 10.1038/s41598-019-42977-2 (PMC6482184; doi:10.1038/s41598-019-42977-2)
Supplement: Supplementary file 1 — Supplementary Data [file 41598_2019_42977_MOESM1_ESM.pdf]

Supplementary Data

Tropomyosin concentration but not formin nucleators mDia1 and mDia3 determines the level of tropomyosin incorporation into actin filaments

Joyce C. M. Meiring[1], Nicole S. Bryce[1], Jorge Luis Galeano Niño[2], Antje Gabriel[1,3], Szun S. Tay[2], Edna C. Hardeman[1], Maté Biro[2], Peter W. Gunning[1*]

[1]Cellular and Genetic Medicine Unit, School of Medical Sciences, University of New South Wales, Sydney, NSW 2052, Australia.

[2]Single Molecule Science, School of Medical Sciences, University of New South Wales, Sydney, NSW 2052, Australia.

[3]Pharmaceutical Biology, Center for Drug Research, Ludwig-Maximilians-Universität, Munich, Germany.

*Corresponding Author: ORCID: 0000-0003-0833-3128; email: p.gunning@unsw.edu.au; mailing address: School of Medical Sciences, UNSW Australia, Sydney, NSW 2052, Australia.

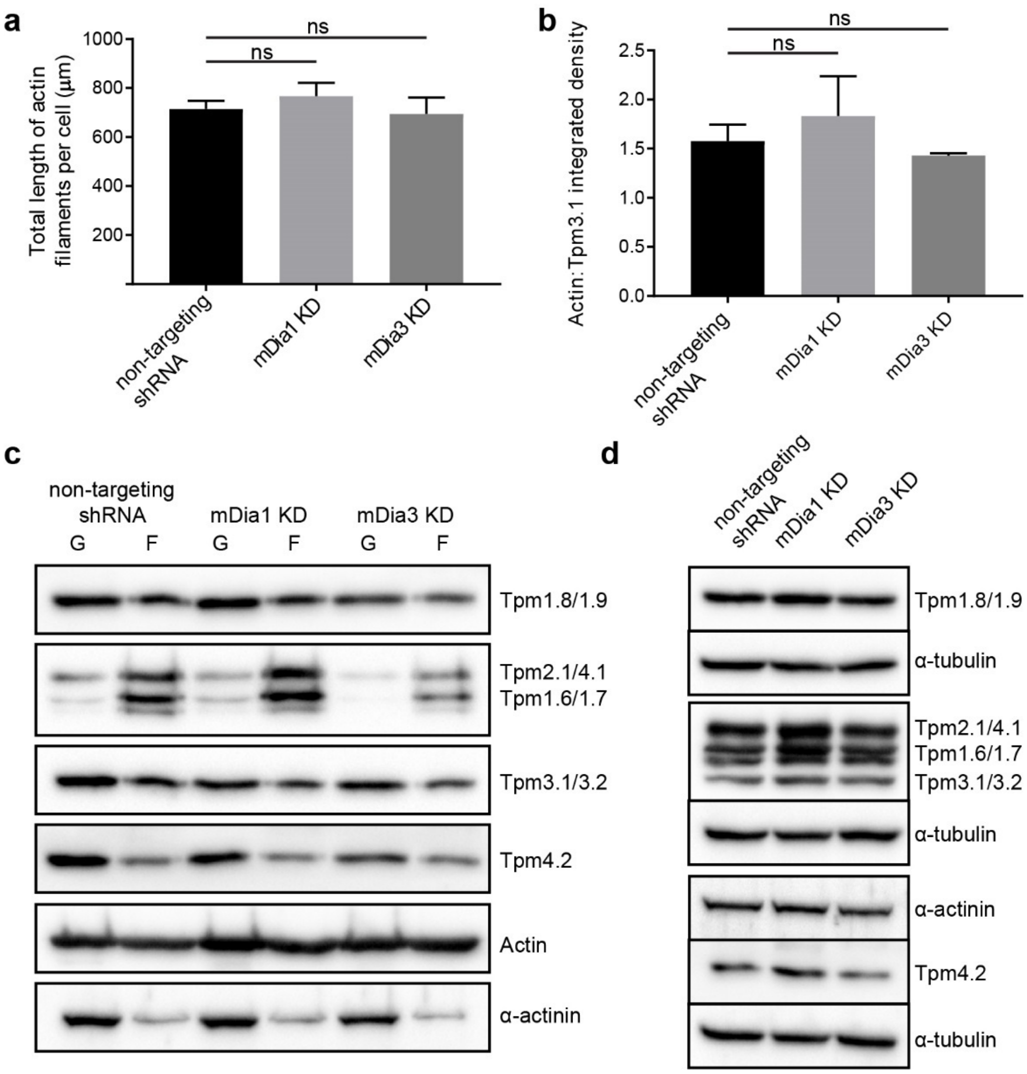

Supplementary Figure S1. Knockdown of mDia1 and mDia3 KD does not alter tropomyosin function or levels within the cell. (**a**) Histogram showing the total length of actin stress fibres per cell, presented as mean ± SD, quantified from 3 experiments, ns = not significant. (**b**) Histogram of the ratio of fluorescence intensity of actin to Tpm3.1, presented as mean ± SD, quantified from 3 experiments, ns = not significant. (**c**) Partitioning of tropomyosins, actin and α-actinin with F-actin pellet was measured via a biochemical assay in mDia1 and mDia3 KD cells as well as non-targeting shRNA expressing cells. (**d**) Protein levels of tropomyosins and α-actinin were measured via western blotting and compared between mDia1 KD, mDia3 KD and non-targeting shRNA expressing BJeH cells. Representative blots are shown, refer to Figure 1f and 1g for graphed data.

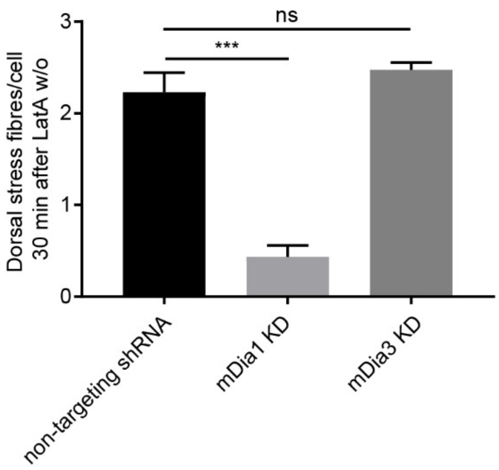

Supplementary Figure S2. Knockdown of mDia1 but not mDia3 results in a loss of dorsal stress fibres in cells 30 min after the washout of LatA. Histogram of the number of dorsal stress fibres per cell presented as mean ± SD from 3 experiments, *** P<0.001, ns=not significant.

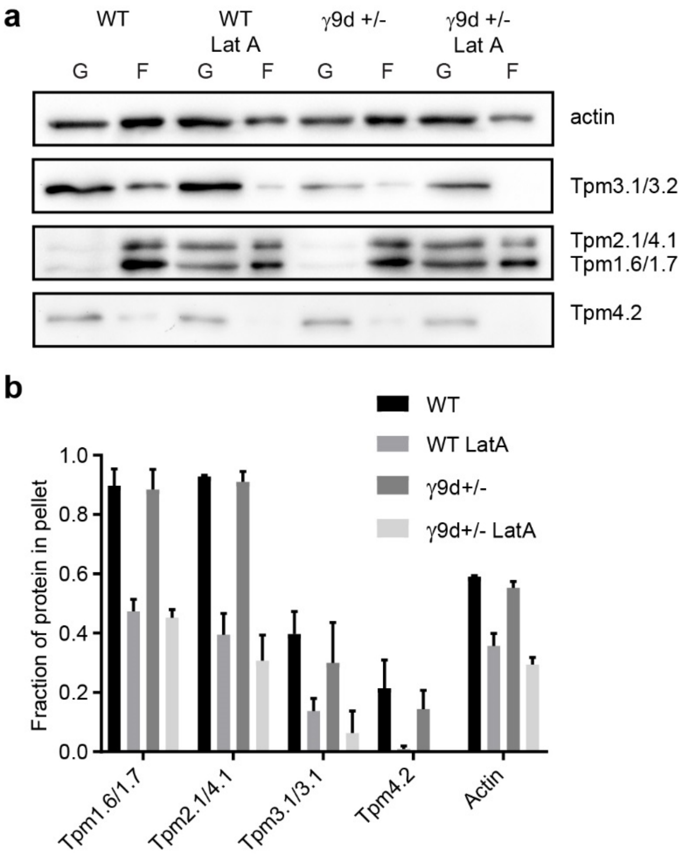

Supplementary Figure S3. Tpm3.1 recruitment to actin filaments is dependent on intact actin filaments. Partitioning of tropomyosins and actin with F-actin pellet was measured via a biochemical assay in WT and γ9d +/- MEFs that were treated with either DMSO or 5 µM LatA. (**a**) Representative blots and (**b**) Quantification across 3 independent experiments presented as mean ± SD. All LatA treatments were significantly different from vehicle treated controls in both cell types ($P<0.001$), whereas there was no significant difference detected between cell types that were treated under the same conditions (DMSO or LatA).

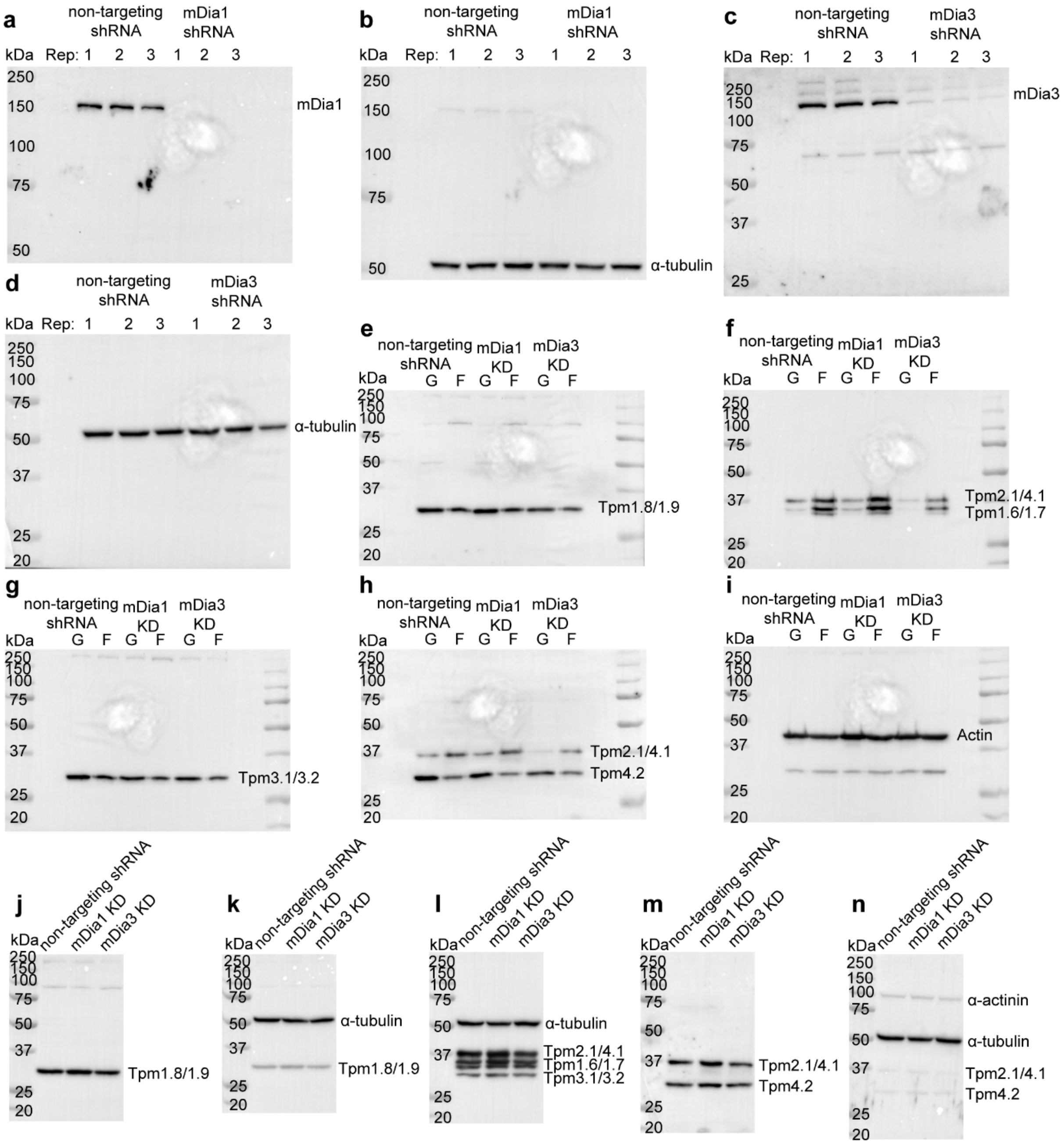

Supplementary Figure S4. Full length blots of the cropped Westerns in Fig 1 and Fig S1. a and b refer to Fig 1b, c and d refer to Fig 1c. d-i refer to Fig 1f and Fig S1a. j-n refer to Fig 1g and FigS1b.

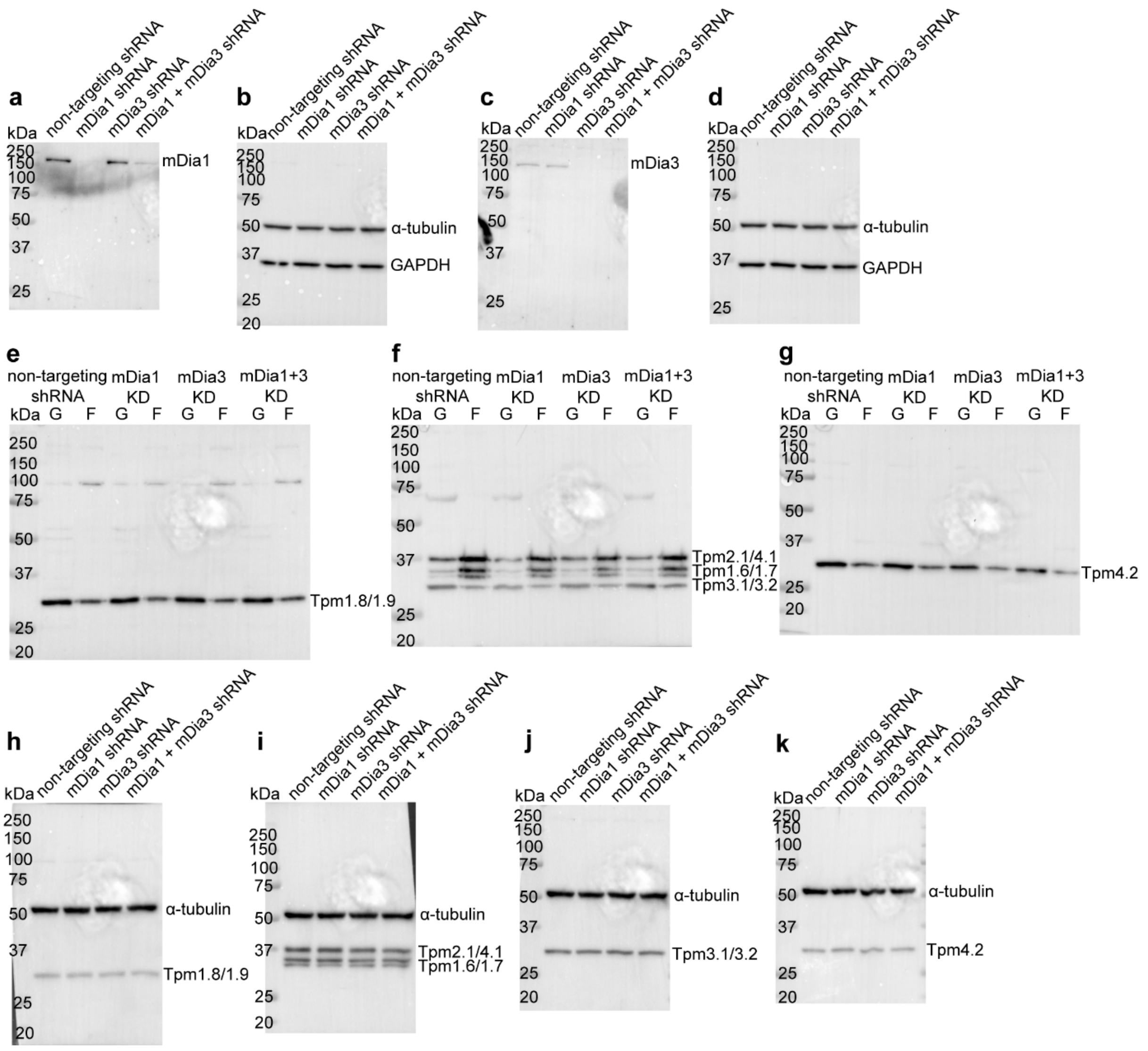

Supplementary Figure S5. Full length blots of the cropped Westerns in Fig 4. a-d refer to Fig 4a, e-g refer to Fig 4d, h-k refer to Fig 4e.

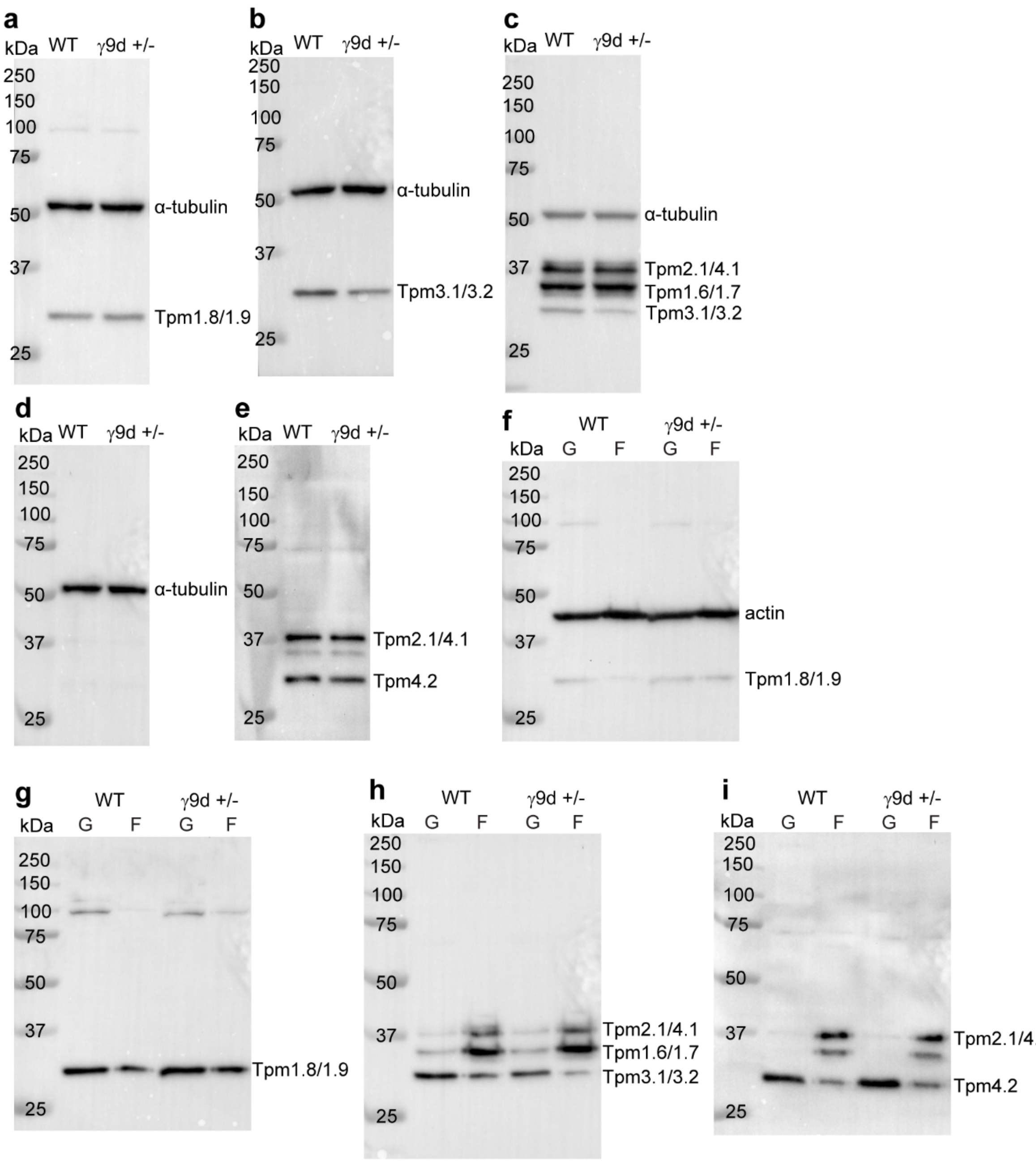

Supplementary Figure S6. Full length blots of cropped Westerns in Fig 5. a-e refer to Fig 5b, f-i refer to Fig 5d.

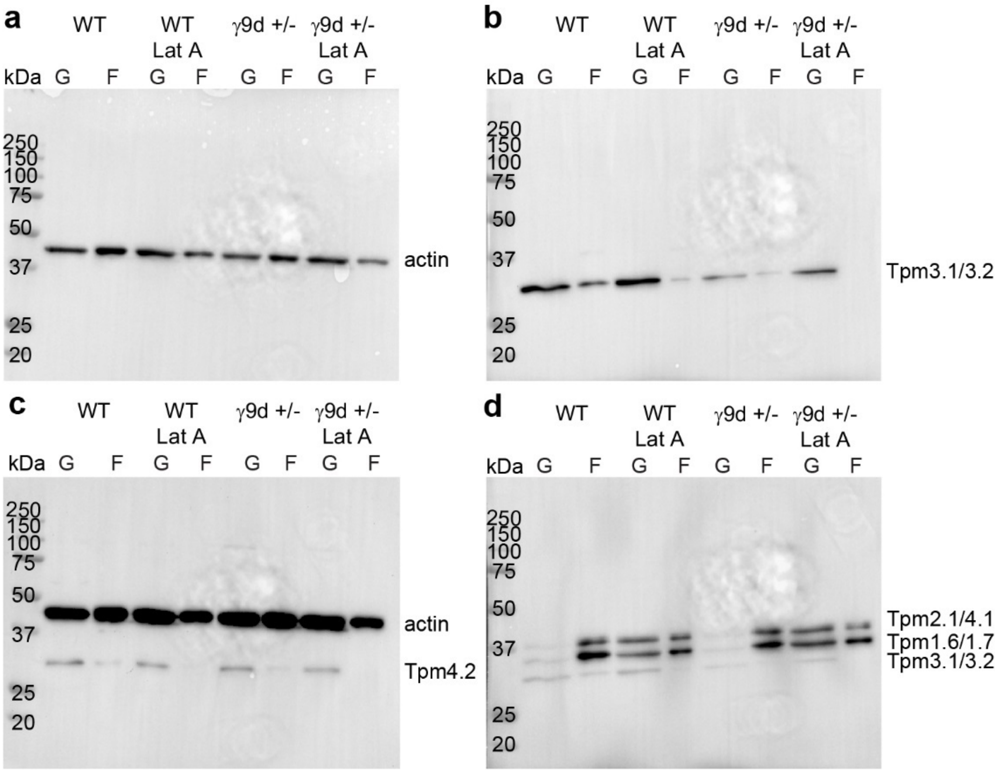

Supplementary Figure S7. Full length blots of cropped Westerns in Supplementary Figure S3.
